# Prevalence and genotype distribution of human papillomavirus infection among women in Chengdu, China between 2020 and 2024

**DOI:** 10.3389/fpubh.2025.1740177

**Published:** 2026-01-05

**Authors:** Wenjuan Wang, Junying Zhang, Chaorong Mei, Qianling Dai, Yiling Cai, Xiaoqing Wei, Yan Li

**Affiliations:** 1 Department of Clinical Laboratory, Chengdu Women’s and Children’s Central Hospital, School of Medicine, University of Electronic Science and Technology of China, Chengdu, China; 2 Department of Oncology, Hospital of Chengdu Office of People's Government of Xizang Autonomous Region (Hospital. C.X.), Chengdu, China; 3 Department of Cervical Disease and Cervical Cancer Prevention and Treatment, Chengdu Women’s and Children’s Central Hospital, School of Medicine, University of Electronic Science and Technology of China, Chengdu, China; 4 Gynecology, Chengdu Women’s and Children’s Central Hospital, School of Medicine, University of Electronic Science and Technology of China, Chengdu, China; 5 Obstetrics, Chengdu Women’s and Children’s Central Hospital, School of Medicine, University of Electronic Science and Technology of China, Chengdu, China

**Keywords:** cervical cancer prevention, Chengdu, epidemiology, genotype, human papillomavirus, prevalence

## Abstract

**Background:**

Cervical cancer remains a significant threat to women’s health globally. This study aimed to investigate the epidemiological characteristics of human papillomavirus (HPV) infection among women in Chengdu, China, between 2020 and 2024, to provide localized evidence for guiding cervical cancer prevention and control strategies.

**Methods:**

A retrospective analysis was conducted using HPV screening data from 51,556 women at Chengdu Women’s and Children’s Central Hospital (CWCCH) between September 2020 and December 2024. Polymerase chain reaction-gene chip technology (PCR-GCT) was used to genotype 26 HPV types (17 high-risk [HR] and 9 low-risk [LR]). Statistical analyses included the chi-square test and Cochran-Armitage trend analysis.

**Results:**

The overall HPV prevalence was 22.03% (11,360/51,556). The infection rate was significantly higher among gynecological outpatients (25.46%) than among participants undergoing routine health screening (13.97%). Single infections predominated (76.47%). The most prevalent HR-HPV genotypes were HPV-52 (3.89%), −16 (3.11%), −58 (2.58%), −51 (2.17%), and −39 (1.54%). A significant increasing trend in prevalence was observed from 2020 (18.70%) to 2024 (25.25%) (*p* < 0.001). Age-specific prevalence showed a bimodal distribution, with the first peak in the ≤20 age group (49.35%) and a second smaller peak in the ≥61 age group (30.79%). Prevalence in spring, summer, and autumn was significantly higher than in winter. Notably, 39.40% of LR HPV infections involved co-infections with HR types.

**Conclusion:**

The high and increasing HPV prevalence in Chengdu, along with its bimodal age distribution, seasonal variation, and frequent HR-LR co-infections—highlights the need for targeted interventions. HPV genotyping is recommended for patients with genital warts, particularly prior to procedures such as excision, to identify high-risk co-infections. A dual strategy of “vaccination and standardized screening” should be reinforced, including promoting the 9-valent vaccine (covering HPV-16/52/58) among young women and enhanced systematic screening for middle-aged and older women to control the second infection peak.

## Introduction

Human papillomavirus (HPV) is a non-enveloped, double-stranded circular DNA virus with a genome size of approximately 8 kb. It belongs to the Papillomaviridae family and Alphapapillomavirus genus. As one of the most common sexually transmitted infections, HPV enters through minor abrasions in the skin or mucosa during sexual contact and infects basal epithelial cells (1). Approximately 70% of sexually active women will acquire an HPV infection during their lifetime (2). Fortunately, most cervical HPV infections—whether accompanied by cytological abnormalities or not—are cleared or suppressed by the cellular immune response within 1 to 2 years after initial exposure (3). However, persistent infection occurs in about 10% of cases, significantly increasing the risk of developing precancerous lesions (4). Not all HPV genotypes are carcinogenic. Low-risk HPV types (LR-HPVs) primarily cause benign genital warts and skin lesions, whereas high-risk HPVs (HR-HPVs) can lead to oropharyngeal cancers (affecting the oral cavity, tonsils, and throat) and various anogenital cancers, including cervical, anal, vulvar., vaginal, and penile cancers. Specifically, 15 genotypes—16, 18, 31, 33, 35, 39, 45, 51, 52, 56, 58, 59, 68, 73, and 82—are classified as high-risk (HR), while 12 genotypes—6, 11, 40, 42, 43, 44, 54, 61, 70, 72, 81, and CP6108—are considered low-risk (LR) (5). The carcinogenic mechanism of HPV is primarily associated with two oncoproteins it encodes, E6 and E7. These two proteins work synergistically to promote cell proliferation, extend the cell life cycle, and prevent apoptosis. This ultimately leads to uncontrolled cell division and the accumulation of genetic damage (6). Persistent infection with high-risk HPV (HR-HPV) is strongly associated with the development of cervical cancer, accounting for approximately 98.7% of cases (7). Among these, HPV-16 and HPV-18 are the most carcinogenic types, responsible for about 70% of cervical cancer cases and 50% of cervical intraepithelial neoplasia grade 3 (CIN3) cases (8). In contrast, low-risk (LR) HPV-6 and HPV-11 cause approximately 90% of genital wart cases.

The HPV is a necessary but not sufficient cause of cervical cancer (2). Other significant co-factors include certain sexually transmitted infections (such as HIV and *Chlamydia trachomatis*), smoking, multiple childbirths, and long-term use of oral contraceptives (9). Globally, cervical cancer ranks as the fourth most common cancer and the fourth leading cause of cancer-related deaths among women globally, posing a major public health challenge. According to recent global statistics, there are approximately 604,000 new cases and 342,000 deaths annually, with the majority occurring in sub-Saharan Africa, Melanesia, South America, and Southeast Asia (10). Nearly 70% of the global cervical cancer burden is observed in less developed countries. As a developing country, China reports around 109,741 new cervical cancer cases and approximately 59,060 deaths each year, accounting for 18.2% of global new cases and 17.3% of global deaths, respectively (11). Cervical cancer is not only one of the most frequent and severe cancers among women in many developing regions, but its societal impact is further exacerbated by the relatively young average age at death—often during child-rearing years. Moreover, cases are frequently diagnosed at advanced stages due to inadequate or absent screening programs. Therefore, eliminating cervical cancer remains a critical global public health challenge.

Cervical cancer is a highly preventable and, if detected early, readily treatable disease. However, it remains a persistent major challenge in low- and middle-income countries that lack systematic screening and prevention programs. There is robust evidence that HPV vaccination is not only effective but also cost-effective (12–14). Nevertheless, various obstacles hinder the promotion and implementation of these measures across different countries (15). China, for instance, introduced the HPV vaccine relatively late and has not yet included it in the national immunization program. Data from 2021 show that the HPV vaccination rate among females nationwide was only 3%, with just 1.9% of girls aged 9–14 vaccinated (16). In response to the World Health Organization’s (WHO’s) global call to eliminate cervical cancer, many regions in China are implementing free HPV vaccination programs under the “Healthy China 2030” initiative. These efforts currently focus on specific groups, such as 13–14-year-old girls in Chengdu, with plans to gradually expand coverage. Despite this, economic and psychosocial factors continue to hinder broader vaccine uptake (16).

In the realm of screening, HPV testing allows for the screening interval to be extended to up to 5 years or longer, owing to its high sensitivity and stable negative predictive value. Therefore, the WHO recommends it as the primary screening method (15). Despite continuous improvements in China’s cervical cancer screening system, the coverage rate remains low. The screening rate for Chinese women aged 35–44 is only 43.4% (17), which falls far short of the WHO’s 70% target (18).

Cervical cancer is a malignant tumor that poses a serious threat to women’s health and lives globally. Effective control and prevention measures are imperative both worldwide and in China. Against this backdrop, HPV genotyping data in Chengdu region are extremely scarce. As the largest women and children’s medical center in southwestern China, CWCCH has a service network covering the entire Chengdu area and a wide range of patient sources. The prevalence of HPV diseases in this hospital is highly representative of the region. Therefore, this study is based on the HPV screening data of 51,556 women at this hospital, aiming to provide more targeted strategies for the prevention, treatment, and eventual elimination of cervical cancer and HPV-related diseases.

## Materials and methods

### Study population

Patients sought HPV screening at CWCCH for various reasons, primarily including health check-ups, patient self-request, diagnostic needs indicated by physicians based on clinical symptoms (such as abnormal vaginal bleeding, lower reproductive tract inflammation, genital warts, unexplained lower abdominal pain, urethritis, etc.), and routine screening in the management of gynecological conditions (such as infertility and gynecological tumors). The inclusion criteria for the study were women with a history of sexual activity who were non-menstruating and not pregnant. The exclusion criteria included no history of sexual activity, being menstruating or pregnant, and those who had undergone hysterectomy. This criterion ensures precise focus on the core at-risk population while guaranteeing that cervical specimens are free from contamination by menstrual blood or physiological changes during pregnancy, thereby maintaining detection quality and comparability. However, the findings cannot be directly generalized to women without sexual history, pregnant women, or those who have undergone hysterectomy, which represents a major limitation of this study. For cases with multiple screenings, only the initial screening results were included. During the defined study period, a total of 67,435 screening records were collected. After excluding 15,879 cases according to the above criteria, this study ultimately included 51,556 participants. The study period spanned from September 2020 to December 2024. The mean age of the participants was 36.2 ± 7.6 years, with an age range of 15–93 years. For analysis, participants were categorized into the following age groups: ≤20 years, 21–30 years, 31–40 years, 41–50 years, 51–60 years, and ≥61 years. This study was approved by the Medical Ethics Committee of CWCCH, and all methods were performed in accordance with relevant guidelines and regulations.

### Ethics statement

As this study is retrospective in nature and all patient identifiers have been deliberately anonymized, obtaining informed consent was not required. The study protocol was approved by the Medical Ethics Committee of CWCCH, with a waiver of informed consent.

### Study methods

#### Specimen collection

Gynecologists collected cervical cell samples using a cervical brush. The brush was inserted into the cervical os and rotated five times clockwise to ensure a sufficient yield of exfoliated epithelial cells. Prior to this step, any excess cervical secretions were cleared using a cotton swab if necessary. The brush head was then detached and placed into a specimen tube labeled with the patient’s identifier. All tubes were securely sealed and transported to the laboratory promptly in accordance with standard testing protocols.

### DNA extraction and HPV genotyping

This assay utilizes an HPV genotyping kit (Shenzhen GL Biotechnology Co., Ltd.) approved by the China Food and Drug Administration (CFDA). Based on gene chip technology (GCT) (19), it detects 26 HPV genotypes, including 17 high-risk types (16, 18, 31, 33, 35, 39, 45, 51, 52, 53, 56, 58, 59, 66, 67, 68, 73) and 9 low-risk types (6, 11, 40, 42, 43, 44, 54, 55, 57). The detection process consists of four sequential steps: (1) HPV-DNA extraction, (2) PCR amplification, (3) DNA hybridization, and (4) detection and analysis.

(1) HPV-DNA extraction involves sample centrifugation, addition of extraction buffer followed by boiling, and a subsequent centrifugation to obtain the DNA supernatant.

(2) PCR amplification is performed using the Haema9600 instrument (Zhuhai XZ Biotechnology Co., Ltd.). The process includes preparing and aliquoting the PCR reaction mixture, adding the test DNA sample, and conducting amplification under the following program: 50 °C for 2 min; 95 °C for 10 min; 40 cycles of denaturation at 95 °C for 30 s, annealing at 52 °C for 45 s, and extension at 65 °C for 30 s; and a final extension at 65 °C for 5 min.

(3) DNA hybridization is carried out using the GL-HB-9600 system (Shenzhen GL Biotechnology Co., Ltd.). The procedure includes positioning the chip in a 96-well plate, pre-hybridization with Wash Buffer B at 55 °C, mixing the PCR product with denaturation buffer, hybridizing the denatured product with hybridization neutralization buffer at 55 °C, and completing the hybridization with three washes using Wash Buffer B.

(4) Detection and analysis is performed using the HPV-GenoCam-9600 gene chip detection and reading system (Shenzhen GL Biotechnology Co., Ltd.). After color development, positive results are displayed as distinct blue dots. The assay incorporates two internal control points, one negative positioning point, and three positive positioning points. The entire test result is considered invalid if any internal control point fails. Performance validation has confirmed no cross-reactivity with common genital bacteria, viruses, or among the 26 HPV genotypes tested.

### Statistical analysis

Data organization and preliminary analysis were performed using Microsoft Excel 2021. All statistical analyses were conducted with SPSS 26 software (SPSS Inc., Chicago, IL, United States), including the chi-square test for comparing sample rates between groups and gamma-distributed linear regression models for analyzing temporal trends in genotype-specific prevalence. Results are presented with gamma values, *p*-values, and 95% confidence intervals, with a two-sided *p*-value < 0.05 considered statistically significant.

## Results

### Overall HPV infection prevalence

A total of 51,556 women in the Chengdu region were tested for 26 HPV genotypes between 2020 and 2024. The overall positive infection rate was 22.03% (11,360/51,556; 95%CI, 21.68–22.39). Further analysis revealed that the HPV infection rate among gynecological outpatients was 25.46% (9,214/36,190), which was significantly higher than the 13.97% (2,146/15,366) observed in the routine health screening group (*χ*^2^ = 829.512, *p* < 0.001).

**Figure 1:**
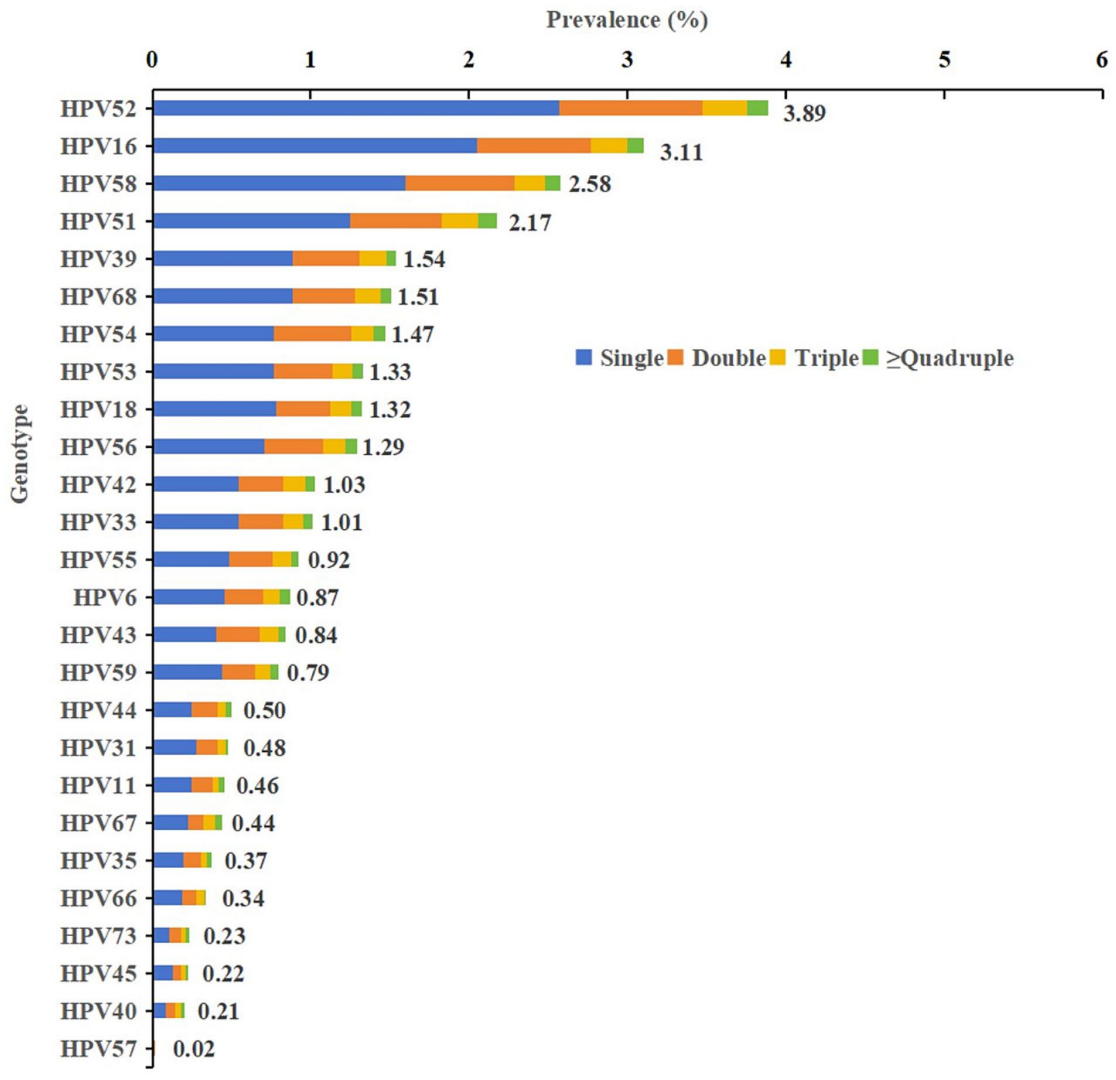
Prevalence and genotype distribution of 26 – genotype infections (including infection patterns) among 51,556 females in Chengdu, China, 2020–2024.

**Figure 2:**
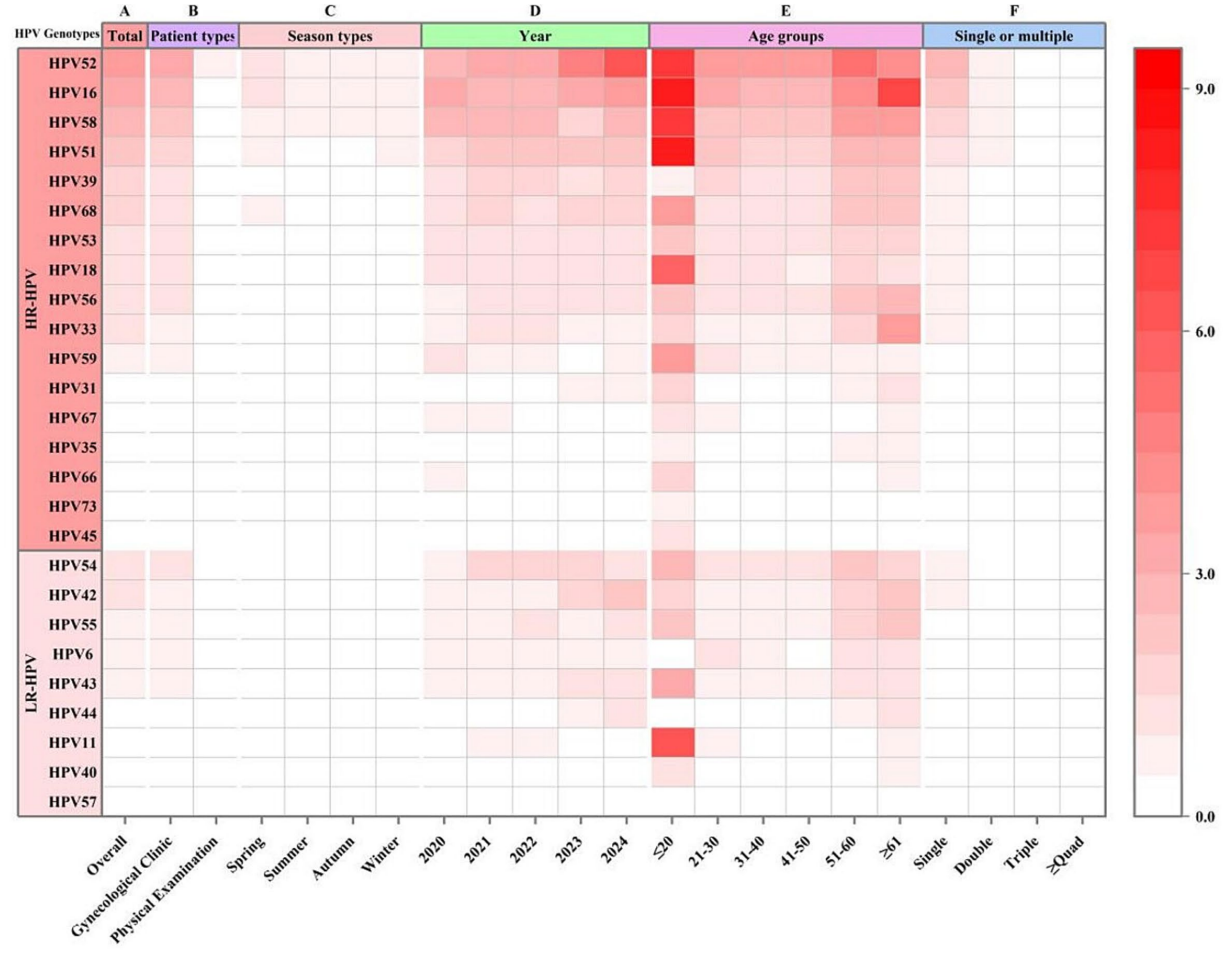
The distribution of 26 HPV genotypes in different grouping modes among 51,556 females in Chengdu, China, 2020–2024.

From a genotype perspective, a total of 14,935 positive genotypic detections were recorded across all 26 genotypes, yielding an overall genotypic positive rate of 28.97% (14,935/51,556; 95%CI, 28.58–29.36). HR genotypes accounted for 22.65% (11,680 detections), and LR genotypes accounted for 6.31% (3,255 detections) (Figures 1, 2 and Tables 1–4).

**Table 1:** Prevalence of infection types and patterns groups by year among 51,556 females in Chengdu, China, 2020–2024.

HPV infection	Positive cases	% (95% CI) for all samples	2020 (*n* = 4,583)	2021 (*n* = 15,961)	2022 (*n* = 13,562)	2023 (*n* = 10,562)	2024 (*n* = 6,888)	*χ* ^2^	*P*
Positive cases	11,360	22.03 (21.68–22.39)	857 (18.70)	3,487 (21.85)	2,933 (21.63)	2,344 (22.19)	1739(25.25)	72.838	<0.001
Infection types									
Single HR	7,014	13.60(13.31–13.90)	556(12.13)	2,160(13.53)	1846(13.61)	1,447(13.70)	1,005(14.59)	14.307	0.006
Single LR	1,673	3.25(3.09–3.40)	109(2.38)	495(3.10)	428(3.16)	378(3.58)	263(3.82)	23.315	<0.001
HR + HR	1,366	2.65(2.51–2.79)	116(2.53)	434(2.72)	340(2.51)	254(2.40)	222(3.22)	12.851	0.012
HR + LR	1,174	2.28 (2.15–2.41)	69 (1.51)	363(2.27)	284(2.09)	235(2.22)	223(3.24)	42.982	<0.001
LR + LR	133	0.26(0.21–0.30)	7(0.15)	35(0.22)	35(0.26)	30(0.28)	26(0.38)	7.002	0.136
Infection pattern									
Single	8,687	16.85(16.53–17.17)	665(14.51)	2,655(16.63)	2,274(16.77)	1825(17.28)	1,268(18.41)	31.838	<0.001
Double	2000	3.88(3.71–4.05)	131(2.86)	617(3.87)	501(3.69)	396(3.75)	355(5.15)	44.554	<0.001
Triple	501	0.97(0.89–1.06)	44(0.96)	161(1.01)	120(0.88)	95(0.90)	81(1.18)	4.856	0.302
≥Quad	172	0.33(0.28–0.38)	17(0.37)	54(0.34)	38(0.28)	28(0.27)	35(0.51)	9.167	0.057

**Table 2:** Prevalence of HPV infection types, patterns and years by age among 51,556 females in Chengdu, China, 2020–2024.

HPV infection	Positive cases	% (95% CI) for all samples	≤20 (*n* = 310)	21–30 (*n* = 16,959)	31–40 (*n* = 20,491)	41–50 (*n* = 7,680)	51–60 (*n* = 4,885)	≥61 (*n* = 1,231)	*χ* ^2^	*P*
Positive cases	11,360	22.03(21.68–22.39)	153(49.35)	3,648(21.51)	4,197(20.48)	1,544(20.10)	1,439(29.46)	379(30.79)	394.387	<0.001
Infection pattern										
Single	8,687	16.85(16.53–17.17)	84(27.10)	2,700(15.92)	3,337(16.29)	1,256(16.35)	1,066(21.82)	244(19.82)	133.644	<0.001
Double	2000	3.88(3.71–4.05)	40(12.90)	687(4.05)	696(3.40)	232(3.02)	260(5.32)	85(6.90)	154.528	<0.001
Triple	501	0.97(0.89–1.06)	16(5.16)	192(1.13)	134(0.65)	48(0.63)	77(1.58)	34(2.76)	151.726	<0.001
≥Quad	172	0.33(0.28–0.38)	13(4.19)	69(0.41)	30(0.15)	8(0.10)	36(0.74)	16(1.30)	233.859	<0.001
Infection types										
Single HR	7,014	13.60(13.31–13.90)	52(16.77)	2,153(12.70)	2,738(13.36)	1,019(13.27)	857(17.54)	195(15.84)	86.064	<0.001
Single LR	1,673	3.25(3.09–3.40)	32(10.32)	547(3.23)	599(2.92)	237(3.09)	209(4.28)	49(3.98)	75.592	<0.001
HR + HR	1,366	2.65(2.51–2.79)	28(9.03)	491(2.90)	444(2.17)	155(2.02)	184(3.77)	64(5.20)	137.965	<0.001
HR + LR	1,174	2.28(2.15–2.41)	37(11.94)	410(2.42)	375(1.83)	118(1.54)	171(3.50)	63(5.12)	246.287	<0.001
LR + LR	133	0.26(0.21–0.30)	4(1.29)	47(0.28)	41(0.20)	15(0.20)	18(0.37)	8(0.65)	26.589	<0.001
Years										
2020	857	1.66(1.55–1.77)	14(4.52)	278 (1.64)	332 (1.62)	130 (1.69)	83 (1.70)	20 (1.62)	23.467	<0.001
2021	3,487	6.76(6.55–6.98)	49(15.81)	1,235 (7.28)	1,292 (6.31)	447 (5.82)	370 (7.57)	94 (7.64)	860.328	<0.001
2022	2,933	5.69(5.49–5.89)	34(10.97)	993 (5.86)	1,119 (5.46)	378 (4.92)	327 (6.69)	82 (6.66)	724.729	<0.001
2023	2,344	4.55(4.37–4.73)	30(9.68)	675 (3.98)	821 (4.01)	372 (4.84)	343 (7.02)	103 (8.37)	230.373	<0.001
2024	1739	3.37(3.22–3.53)	26(8.39)	467 (2.75)	633 (3.09)	217 (2.83)	316 (6.47)	80 (6.50)	199.668	<0.001

**Table 3:** Prevalence of 26 HPV genotypes by year among 51,556 females in Chengdu, China, 2020–2024.

HPV genotype	Positive cases	% (95% CI) for all samples	2020 (*n* = 4,583)	2021 (*n* = 15,961)	2022 (*n* = 13,562)	2023 (*n* = 10,562)	2024 (*n* = 6,888)	*χ* ^2^	*P*	Gamma value	Trend
Total	14,935	28.97(28.58–29.36)	1,133(24.72)	4,599(28.81)	3,801(28.03)	3,022(28.61)	2,380(34.55)	151.24	<0.001	0.061	Increasing
HR-HPV											
52	2006	3.89(3.72–4.06)	117(2.55)	525(3.29)	450(3.32)	493(4.67)	421(6.11)	157.206	<0.001	0.185	Increasing
16	1,601	3.11(2.96–3.26)	149(3.25)	474(2.97)	373(2.75)	352(3.33)	253(3.67)	16.172	0.003	0.041	Increasing
58	1,330	2.58(2.44–2.72)	129(2.81)	466(2.92)	359(2.65)	187(1.77)	189(2.74)	36.849	<0.001	−0.073	Decreasing
51	1,121	2.17(2.05–2.30)	78(1.70)	327(2.05)	315(2.32)	256(2.42)	145(2.11)	10.64	0.031	0.049	Increasing
39	794	1.54(1.43–1.65)	57(1.24)	251(1.57)	231(1.70)	144(1.36)	111(1.61)	7.555	0.109	0.009	
68	779	1.51(1.41–1.62)	52(1.13)	247(1.55)	188(1.39)	165(1.56)	127(1.84)	11.236	0.024	0.058	Increasing
53	687	1.33(1.23–1.43)	49(1.07)	221(1.38)	169(1.25)	150(1.42)	98(1.42)	4.561	0.335	0.031	
18	683	1.32(1.23–1.42)	57(1.24)	238(1.49)	180(1.33)	118(1.12)	90(1.31)	7.108	0.13	−0.044	
56	667	1.29(1.20–1.39)	43(0.94)	199(1.25)	199(1.47)	126(1.19)	100(1.45)	10.199	0.037	0.047	Increasing
33	521	1.01(0.92–1.10)	41(0.89)	182(1.14)	145(1.07)	89(0.84)	64(0.93)	7.2	0.126	−0.05	
59	409	0.79(0.72–0.87)	46(1.00)	137(0.86)	129(0.95)	37(0.35)	60(0.87)	34.597	<0.001	−0.106	Decreasing
31	248	0.48(0.42–0.54)	16(0.35)	77(0.48)	49(0.36)	56(0.53)	50(0.73)	14.888	0.005	0.114	Increasing
67	229	0.44(0.39–0.50)	23(0.50)	90(0.56)	57(0.42)	31(0.29)	28(0.41)	11.335	0.023	−0.133	Decreasing
35	193	0.37(0.32–0.43)	16(0.35)	62(0.39)	49(0.36)	45(0.43)	21(0.30)	1.874	0.759	−0.01	
66	175	0.34(0.29–0.39)	28(0.61)	73(0.46)	32(0.24)	18(0.17)	24(0.35)	29.778	<0.001	−0.229	Decreasing
73	121	0.23(0.19–0.28)	16(0.35)	27(0.17)	25(0.18)	34(0.32)	19(0.28)	10.888	0.028	0.079	Increasing
45	116	0.22(0.18–0.27)	17(0.37)	32(0.20)	30(0.22)	21(0.20)	16(0.23)	5.122	0.275	−0.057	
LR-HPV											
54	759	1.47(1.37–1.58)	44(0.96)	244(1.53)	214(1.58)	160(1.51)	97(1.41)	10.011	0.04	0.028	Increasing
42	529	1.03(0.94–1.11)	23(0.50)	90(0.56)	104(0.77)	167(1.58)	145(2.11)	165.965	<0.001	0.369	Increasing
55	476	0.92(0.84–1.01)	30(0.65)	139(0.87)	141(1.04)	94(0.89)	72(1.05)	7.354	0.118	0.061	
6	448	0.87(0.79–0.95)	34(0.74)	158(0.99)	133(0.98)	62(0.59)	61(0.89)	15.305	0.004	−0.055	Decreasing
43	434	0.84(0.76–0.92)	27(0.59)	129(0.81)	84(0.62)	122(1.16)	72(1.05)	27.595	<0.001	0.124	Increasing
44	256	0.50(0.44–0.56)	12(0.26)	60(0.38)	52(0.38)	59(0.56)	73(1.06)	58.377	<0.001	0.279	Increasing
11	236	0.46(0.40–0.52)	21(0.46)	88(0.55)	72(0.53)	28(0.27)	27(0.39)	13.917	0.008	−0.122	Decreasing
40	106	0.21(0.17–0.24)	7(0.15)	54(0.34)	20(0.15)	8(0.08)	17(0.25)	25.812	<0.001	−0.175	Decreasing
57	11	0.02(0.01–0.03)	1(0.02)	9(0.06)	1(0.01)	0(0.00)	0(0.00)	14.157	0.007	−0.695	Decreasing

**Table 4:** Prevalence of 26 HPV genotypes by age among 51,556 women in Chengdu, China, 2020–2024.

HPV genotype	Positive cases	% (95% CI) for all samples	≤20 (310)	21–30 (16959)	31–40 (20491)	41–50 (7680)	51–60 (4885)	≥61 (1231)	*χ* ^2^	*P*
Total	14,935	28.97(28.58–29.36)	269(86.77)	4,943(29.16)	5,256(25.65)	1897(24.71)	1980(40.53)	590(47.93)	1213.827	<0.001
HR-HPV										
52	2006	3.89(3.72–4.06)	22(7.10)	631(3.72)	755(3.68)	299(3.89)	247(5.06)	52(4.22)	30.274	<0.001
16	1,601	3.11(2.96–3.26)	25(8.06)	544(3.21)	538(2.63)	201(2.62)	209(4.28)	84(6.82)	126.595	<0.001
58	1,330	2.58(2.44–2.72)	22(7.10)	405(2.39)	501(2.44)	173(2.25)	181(3.71)	48(3.90)	65.547	<0.001
51	1,121	2.17(2.05–2.30)	25(8.06)	415(2.45)	404(1.97)	117(1.52)	125(2.56)	35(2.84)	81.736	<0.001
39	794	1.54(1.43–1.65)	3(0.97)	261(1.54)	298(1.45)	107(1.39)	98(2.01)	27(2.19)	13.218	0.021
68	779	1.51(1.41–1.62)	12(3.87)	246(1.45)	284(1.39)	103(1.34)	105(2.15)	29(2.36)	34.943	<0.001
53	687	1.33(1.23–1.43)	7(2.26)	211(1.24)	260(1.27)	90(1.17)	95(1.94)	24(1.95)	22.654	<0.001
18	683	1.32(1.23–1.42)	18(5.81)	254(1.50)	241(1.18)	67(0.87)	89(1.82)	14(1.14)	76.565	<0.001
56	667	1.29(1.20–1.39)	7(2.26)	207(1.22)	221(1.08)	89(1.16)	109(2.23)	34(2.76)	65.903	<0.001
33	521	1.01(0.92–1.10)	6(1.94)	138(0.81)	168(0.82)	73(0.95)	91(1.86)	45(3.66)	138.509	<0.001
59	409	0.79(0.72–0.87)	11(3.55)	170(1.00)	136(0.66)	45(0.59)	36(0.74)	11 (0.89)	48.245	<0.001
31	248	0.48(0.42–0.54)	5 (1.61)	77(0.45)	85 (0.41)	33(0.43)	35(0.72)	13 (1.06)	25.013	<0.001
67	229	0.44(0.39–0.50)	4(1.29)	93 (0.55)	81 (0.40)	19(0.25)	23(0.47)	9(0.73)	19.386	0.002
35	193	0.37(0.32–0.43)	3(0.97)	55(0.32)	66(0.32)	29(0.38)	32(0.66)	8(0.65)	18.395	0.002
66	175	0.34(0.29–0.39)	5(1.61)	66(0.39)	51(0.25)	27(0.35)	18(0.37)	8(0.65)	24.73	<0.001
73	121	0.23(0.19–0.28)	3(0.97)	39(0.23)	46(0.22)	11(0.14)	19(0.39)	3(0.24)	14.934	0.011
45	116	0.22(0.18–0.27)	4(1.29)	25(0.15)	41(0.20)	18(0.23)	22(0.45)	6(0.49)	35.643	<0.001
LR-HPV										
54	759	1.47(1.37–1.58)	9(2.90)	250(1.47)	276(1.35)	101(1.32)	103(2.11)	20(1.62)	21.733	<0.001
42	529	1.03(0.94–1.11)	6(1.94)	168(0.99)	172(0.84)	68(0.89)	86(1.76)	29(2.36)	58.641	<0.001
55	476	0.92(0.84–1.01)	7(2.26)	128(0.75)	161(0.79)	76(0.99)	75(1.54)	29(2.36)	63.531	<0.001
6	448	0.87(0.79–0.95)	30(9.68)	181(1.07)	137(0.67)	35(0.46)	49(1.00)	16(1.30)	315.415	<0.001
43	434	0.84(0.76–0.92)	10(3.23)	164(0.97)	135(0.66)	52(0.68)	60(1.23)	13(1.06)	44.426	<0.001
44	256	0.50(0.44–0.56)	1(0.32)	72(0.42)	97(0.47)	30(0.39)	40(0.82)	16(1.30)	30.279	<0.001
11	236	0.46(0.40–0.52)	20(6.45)	100(0.59)	70(0.34)	15(0.20)	21(0.43)	10(0.81)	272.049	<0.001
40	106	0.21(0.17–0.24)	4(1.29)	38(0.22)	29(0.14)	16(0.21)	12(0.25)	7(0.57)	30.452	<0.001
57	11	0.02(0.01–0.03)	0(0.00)	5(0.03)	3(0.01)	3(0.04)	0(0.00)	0(0.00)	3.461	0.629

### The prevalence of single and multiple HPV infections

**Figure 3:**
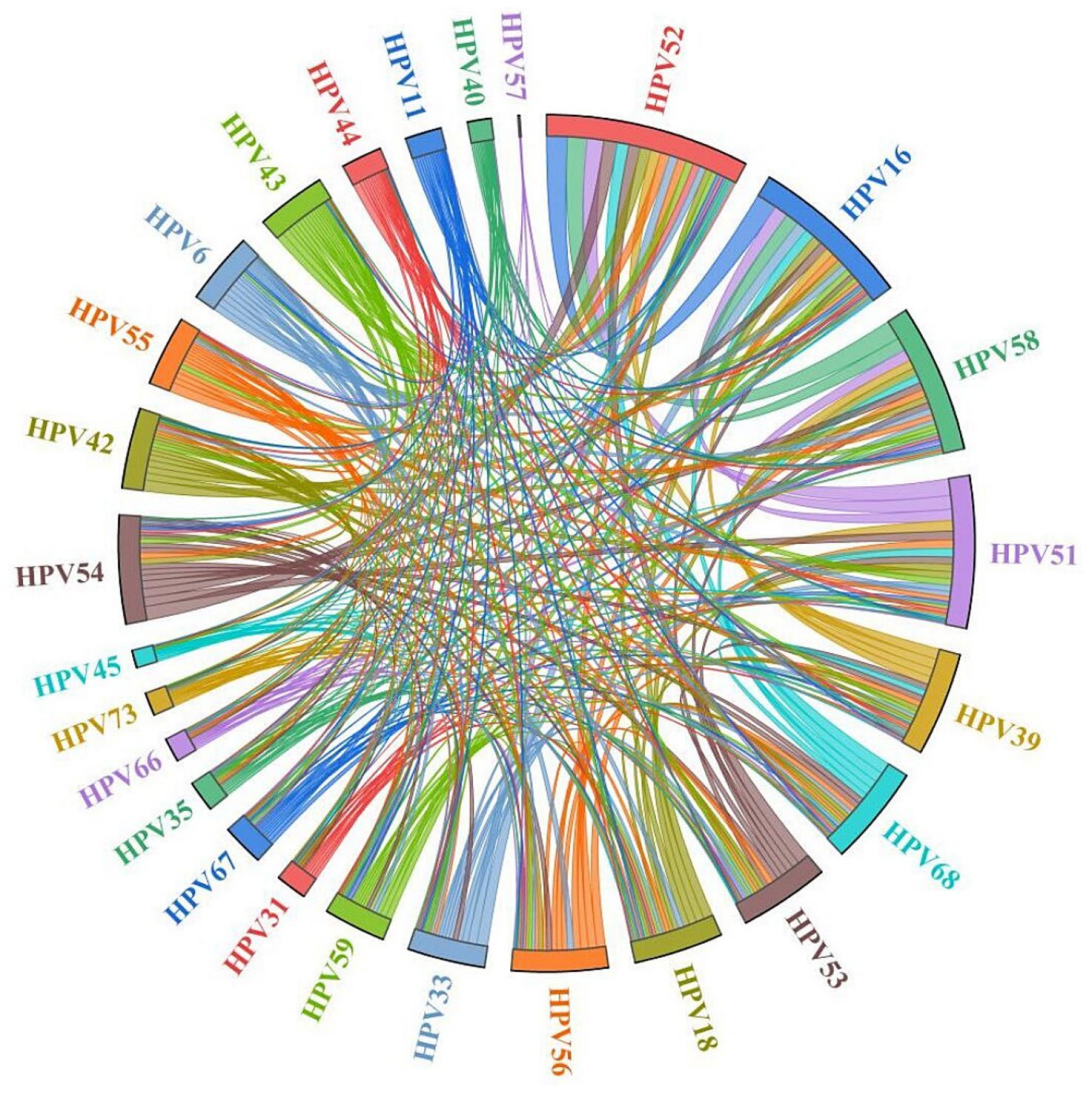
Chord diagram of pairwise HPV type correlations with multiple infections 51,556 females in Chengdu, China, 2020–2024.

Among the 11,360 HPV-positive cases, single infections were predominant (76.47%, 8,687 cases), while multiple infections accounted for 23.53% (2,673 cases). Analysis of multiple infections revealed a declining prevalence with an increasing number of infecting genotypes: double infections were most common (17.61%, 2,000 cases), followed by triple (4.41%, 501 cases) and quadruple or higher-order infections (1.51%, 172 cases). Regarding the composition of co-infections, HR co-infections (12.02%, 1,366 cases) were the most prevalent pattern within multiple infections, significantly outpacing mixed HR-LR co-infections (10.33%, 1,174 cases) and LR co-infections (1.17%, 133 cases). Further analysis showed that among all 9,554 HR-HPV cases, 12.29% (1,174 cases) were HR-LR mixed infections. Conversely, among all 2,980 LR-HPV cases, a substantial proportion of 39.40% (1,174 cases) existed as HR-LR mixed infections, indicating that LR infections are frequently accompanied by HR types. This profile—characterized by the dominance of single infections, a decreasing frequency with increasing infection multiplicity, and the predominance of HR co-infections—remained consistent throughout each year from 2020 to 2024, across all age groups, and was applicable to nearly all HPV genotypes (Figures 1, 2 and Tables 1, 2). The ranking of the 26 genotypes was highly consistent between single and multiple infections, with HPV-52, −16, −58, −51, and −39 consistently occupying the top five positions (Figures 1, 2). Furthermore, results from the circle graph analysis indicated that the most frequent HPV type combinations in multiple infections were: HPV52 with HPV16 (106 occurrences), HPV52 with HPV58 (99 occurrences), HPV52 with HPV51 (85 occurrences), HPV16 with HPV51 (70 occurrences), HPV54 with HPV52 (70 occurrences), and HPV16 with HPV58 (68 occurrences). This finding further confirms that the most common HPV genotypes in multiple infections (such as HPV-52, −16, −58, −51, and −39) were precisely those with the highest overall prevalence; furthermore, the top-ranking LR type, HPV54, also formed its most frequent combination with the highest-prevalence HR type, HPV52 (Figure 3).

### Year and seasonal-specific prevalence of HPV infection

Overall, the prevalence of HPV in Chengdu showed a significant upward trend from 2020 to 2024 (gamma value = 0.061, *p* < 0.001). From 2020 to 2024, significant differences were observed in the prevalence rates of all single infections (including both HR and LR types), double infections, HR co-infections, and mixed HR-LR co-infections (all *p* < 0.05; Tables 1, 3).

**Figure 4:**
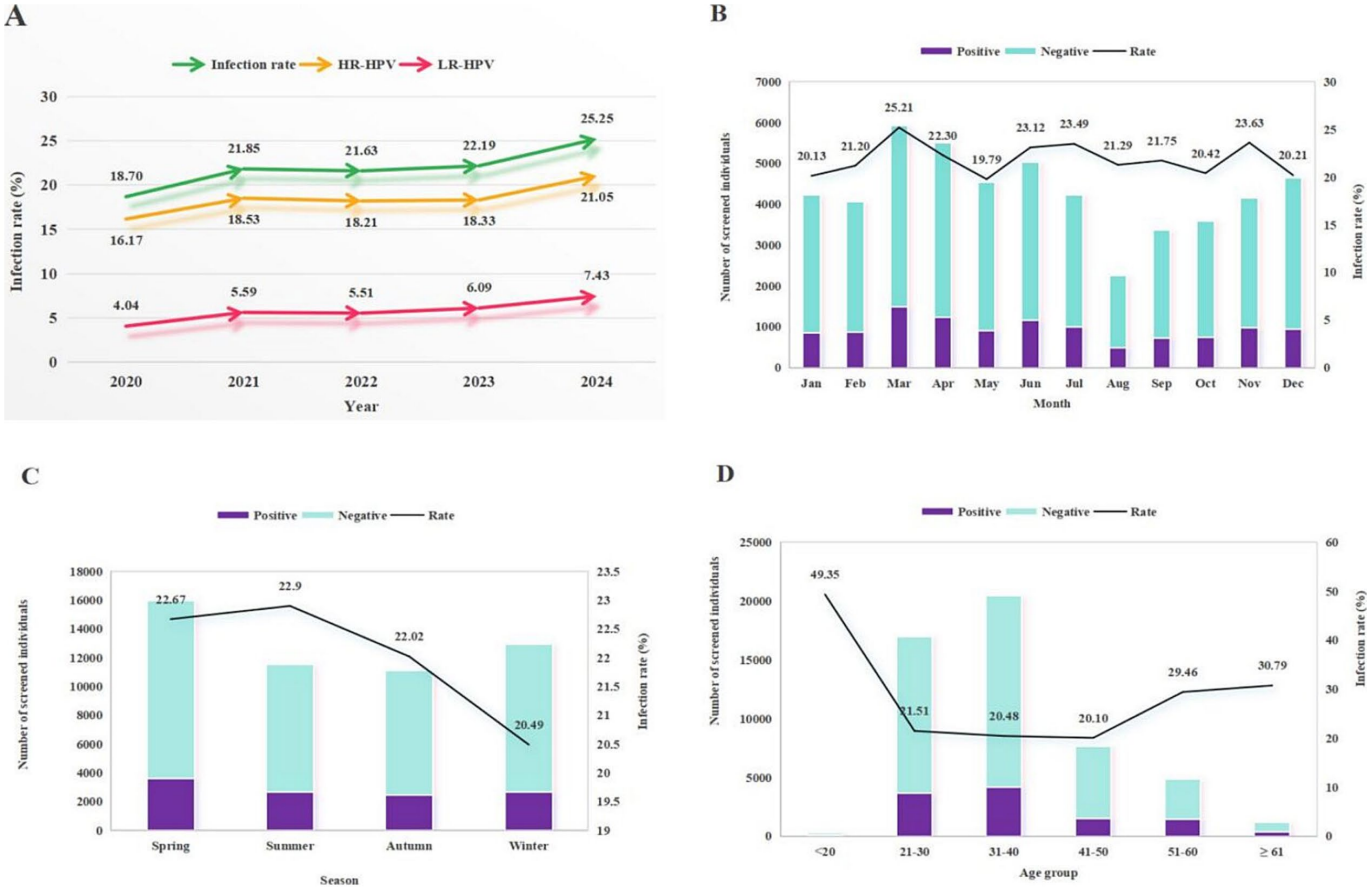
Line plots depicting: **(A)** annual trends in overall HPV, HR-HPV, and LR-HPV prevalence; **(B)** monthly infection rates across 12 months; **(C)** seasonal prevalence (Spring: March–April–May; Summer: June–July–August; Autumn: September–October–November; Winter: December–January–February); **(D)** age-specific infection rates.

The annual prevalence peaked in 2024 (25.25%), in contrast to the nadir observed in 2020 (18.70%) (Tables 1, 3 and Figure 4A). Analysis of monthly distribution revealed the highest infection rate in March (25.21%) and the lowest in May (19.79%). Seasonally, the prevalence in winter (20.49%) was significantly lower than the combined mean prevalence of spring, summer, and autumn (23.17%; *p* < 0.05). No significant differences in prevalence were found among spring, summer, and autumn themselves (*p* > 0.05; Figures 2, 4B,C).

### Age-specific HPV prevalence

Among the 51,556 women who underwent HPV screening, the distribution across age groups was as follows: ≤20 years: 0.60%; 21–30 years: 32.89%; 31–40 years: 39.75%; 41–50 years: 14.90%; 51–60 years: 9.48%; and ≥61 years: 2.39%. Consequently, women aged 21–50 constituted the predominant cohort in this study, whereas the other age groups had comparatively fewer participants (Tables 2, 4).

From 2020 to 2024, the age-specific HPV prevalence demonstrated a bimodal distribution. The first and highest peak was observed in the ≤20 age group (49.35%), followed by a sharp decline. Prevalence remained relatively stable through the 31–50 age groups before rising again, forming a second, smaller peak in the ≥61 age group (30.79%) (Figure 4D). This bimodal pattern was consistently observed across nearly all 26 genotypes, various infection types, various infection patterns, and annually from 2020 to 2024. While the first peak typically involved the ≤20 age group and the second the ≥61 group, the exact age ranges of these peaks varied slightly by genotype and infection category. For instance, HPV-39 and HPV-44 exhibited their first prevalence peak in the 21–30 age group, while HPV-52 and HPV-18 showed their second peak in the 51–60 group. Similarly, the second peak for single infections also occurred in the 51–60 age group, a pattern notably consistent in the 2020 and 2022 data (Figures 2, 4D and Tables 2, 4).

### Type-specific prevalence of HPV infection

Between 2020 and 2024, the study of 51,556 women identified the prevalence of 26 HPV genotypes. The overall HPV infection prevalence demonstrated a significant increasing trend during this period (gamma value = 0.061, *p* < 0.001). HPV-52 was the most prevalent HR genotype (3.89%), followed by HPV-16 (3.11%), HPV-58 (2.58%), HPV-51 (2.17%), and HPV-39 (1.54%). In contrast, several HR genotypes, including HPV-45, −73, −66, −35, −67, −31, and −59, each had a prevalence below 1%. Among LR genotypes, HPV-54, −42, −55, −6, and −43 were the most common, while HPV-11, −40, and −57 were rare, each with a prevalence below 0.50% (Figure 1).

Analysis of temporal trends revealed genotype-specific dynamics. The prevalence of several HR-HPVs (−52, −16, −51,-68, −56, −31, −73) and LR-HPVs (−54, −42, −43,-44) increased significantly over time (gamma value > 0, *p* < 0.05). Conversely, a significant decreasing trend was observed for other genotypes, such as HR-HPVs −58, −59, −66, and −67 and LR-HPVs −6, −11, −40, −57 (gamma value < 0, *p* < 0.05). However, the trends for seven genotypes, including HR-HPVs −39 and −53 and LR-HPVs-55 (increasing) and HR-HPVs −18, −33, −35, and −45 (decreasing), were not statistically significant (gamma value ≠ 0, *p* > 0.05) (Table 3).

Furthermore, the age-specific prevalence of all HPV genotypes except HPV-57 showed statistically significant variations (*p* < 0.05) (Table 4). Differences in genotype prevalence between gynecology and health examination clinics, across seasons, and by infection pattern (single vs. multiple) are visually summarized in the heatmap in Figure 2, with detailed data provided in Figure 1 and Tables 3, 4.

## Discussion

This cohort study, based on 51,556 female HPV screening cases in the Chengdu region from 2020 to 2024, comprehensively analyzed HPV genotype distribution, infection patterns, temporal trends, and age-specific characteristics. HPV prevalence demonstrates significant geographical variation, with a global estimated prevalence of 11.7% (20). The overall HPV prevalence in this study was 22.03%, showing a significant decrease compared to the historical data from our institution covering the eight-year period prior to 2020 (23.28%, *n* = 181,705; *χ*^2^ = 35.065, *p* < 0.001) (21). However, trend analysis revealed an upward trend in the annual infection rate between 2020 and 2024, driven particularly by increases in high-risk genotypes such as HPV-16, HPV-31, HPV-51, HPV-52, HPV-56, HPV-68, and HPV-73.

Geographically, the prevalence rate found in this study was comparable to that of Zhejiang Province (22.3%) (22), which has a similar level of economic development (*p* > 0.05), but was significantly higher than the overall prevalence among Chinese women (17.70%) (23) and that in more economically developed cities such as Beijing (20.15%) (24), Shanghai (18.81%) (25), and Guangzhou (19.78%) (26) (all *p* < 0.05). These disparities may be attributed to a combination of geographical, socioeconomic, behavioral, and cultural factors. Furthermore, it is important to note that the timeframe of this study (2020–2024) spans the COVID-19 pandemic period. The lower infection rate observed in early 2020 may have been partly attributable to interruptions in women’s HPV screening behaviors and healthcare-seeking patterns due to isolation measures during the pandemic, while the upward trend in subsequent years may include a “catch-up screening” effect. The design of this study does not allow for quantification of this impact, which should be considered as an important contextual background when interpreting temporal trends. The design of this study does not allow for quantification of this impact, which should be considered as contextual background when interpreting temporal trends. As studies have found a strong correlation between HPV prevalence and cervical cancer disease burden (20), the high infection rate and the rising trend of specific HR genotypes in Chengdu constitute a significant public health warning and underscore the substantial challenges that remain in achieving the WHO’s 2030 cervical cancer elimination goal in this region.

This study found that the HPV infection rate among gynecological outpatients (25.46%) was significantly higher than that in individuals undergoing routine health examinations (13.97%) (*p* < 0.05). This finding is consistent with a report from Beijing (21.0% vs. 11.9%) (24), which can be attributed to the presence of genital tract infections or lesions in gynecological outpatients, whereas individuals in the general health screening population typically have normal genital tract conditions. Seasonal changes can influence disease distribution by affecting biological functions such as host susceptibility, metabolism, and inflammatory responses (27). This study is the first to observe a distinct seasonal variation in HPV infection rates: the rates in spring, summer, and autumn were significantly higher than in winter (*p* < 0.05), while no significant differences were observed among the first three seasons (*p* > 0.05). This finding differs from a study conducted in Zhejiang (22), which reported the highest HPV infection rates in winter and spring. The climatic differences during winter between the two regions may be one reason for this discrepancy: lower temperatures in Chengdu may partially inhibit virus survival and transmission. Currently, there is no strong biological evidence to support a clear seasonal pattern in HPV infection. Therefore, this finding should be considered preliminary, requiring further validation across different regions and study designs, and interpreted with caution.

This study identified the most prevalent HR-HPV genotypes in the Chengdu region as HPV-52, HPV-16, HPV-58, HPV-51, and HPV-39. Compared to the genotype distribution from our institution 8 years ago (52, 16, 58, 53, 51) (21), four genotypes (52, 16, 58, 51) remained consistent, with only genotype 39 replacing 53, reflecting minor shifts within an overall stable local HPV genotype distribution. Horizontal comparison revealed that the dominant genotype profile in this study was completely consistent with findings from Beijing (52, 16, 58, 51, 39) (24), but differed from the large-scale data across 29 provinces in China (52, 16, 53, 58, 51) (28), as well as studies from Shanghai (52, 16, 58, 53, 39) (25), Guangzhou (52, 58, 16, 51, 39) (26), and Zhejiang (52, 58, CP8304, 16, 51) (22). Comprehensive analysis indicates that HPV-16, HPV-52, and HPV-58 are the dominant viral strains nationwide (21–26, 28). This study further confirms that HPV-52 and HPV-58 are particularly common genotypes in the Asian region. These prevalence disparities reflect both regional epidemiological variations and potential differences in the sensitivity and types of HPV detection kits used for specific genotypes (29).

In terms of carcinogenic risk, HPV-16 carries the highest risk, followed by HPV-18, while other Alpha-9 species members (such as 31, 33, 35, 52, 58) are classified as medium-risk (30). A large-scale study focusing on Chinese patients with invasive cervical cancer (ICC) revealed that the top five HPV types are 16 (62.5%), 18 (12.4%), 58 (8.6%), 52 (5.7%), and 33 (4.6%) (31). The genotype prevalence ranking in our study indicates that HPV-16, 52, and 58 not only have high prevalence rates but also strong carcinogenicity; whereas HPV-18, 31, 33, and 35, despite their relatively lower prevalence, also pose high carcinogenic risks. It is noteworthy that beyond HPV-16 and 18, HPV-52 and 58 collectively account for 13.3% of cervical cancer cases in China (31). Cervical cancer risk is determined by both the specific HPV types involved and insufficient screening coverage. The 33.06% detection rate (4,937 genotype-positive cases) of HPV-16, 52, and 58 identified in this study highlights the crucial importance of implementing targeted vaccination and systematic screening as fundamental strategies for cervical cancer control.

Among the five vaccines currently approved in China (21), only Gardasil® 9 covers types 16, 52, and 58 simultaneously. Promisingly, domestic vaccine development is progressing rapidly: two Shanghai-based institutes have vaccines in clinical trial stages—a non-avalent vaccine (covering types 6, 11, 16, 18, 31, 33, 45, 52, 58) and a quadrivalent vaccine (targeting 16, 18, 52, 58) (32). These candidate vaccines will better meet the protection needs of women in China and other Asian regions, as types 52 and 58 are predominant circulating strains in this area. In summary, among the currently available vaccines, Gardasil® 9 can prevent the three most common HR genotypes (HPV-52, HPV-16, and HPV-58) in this region, and its protective scope is significantly superior to the quadrivalent vaccine, which only covers types 16, 18, 6, and 11. Therefore, Gardasil®9 is currently the option that best aligns with the specific preventive needs of the female population in Chengdu, China.

This study revealed a typical bimodal age-specific distribution of HPV infection rates. The first peak occurred in the ≤20 years age group, with a prevalence rate as high as 49.35%; subsequently, the infection rate gradually declined and stabilized during middle age; and a second, smaller peak emerged in the ≥61 years age group, with a prevalence rate of 30.79%. This bimodal pattern is consistent with the findings of numerous global studies (20–26). The first peak among young women often occurs shortly after sexual debut (around age 15) (33) and is primarily associated with factors such as an immature immune system and relatively high sexual activity (20). It is noteworthy that most infections at this stage are transient; approximately 80–90% are cleared by the body’s immune system within 1 to 2 years (34). To effectively address this early infection peak, completing vaccination before female sexual debut has been proven to be the most cost-effective key strategy – as existing vaccines cannot treat established infections or lesions, and cross-protection effects in vaccinated populations are limited, preemptive prevention is particularly important. A cohort study covering 179 countries and 58 million 12-year-old girls confirmed that vaccination could prevent 690,000 cases of cervical cancer and 420,000 deaths in their lifetime, with a cost per disability-adjusted life year (DALY) averted lower than the per capita GDP, demonstrating favorable health economic benefits (14). A further US study based on health insurance data also showed significantly reduced incidence of genital warts, cervical cytological abnormalities, and high-grade cervical intraepithelial neoplasia (CIN) among vaccinated females aged 14–19 (35), strongly validating the practical effectiveness of this strategy.

To effectively address the second infection peak in middle-aged and older women, it is crucial to continuously promote regular cervical cancer screening. Given that the HPV infection rate in this study stabilized around age 30, and persistent infection is a major risk factor for cervical cancer in women of this age group (36), screening can not only reduce precancerous lesions and clear persistent infections but may also prevent subsequent infections through an antigen presentation effect (37). The WHO’s Cervical Cancer Elimination Strategy sets a 2030 target of achieving 70% high-performance screening coverage for women at ages 35 and 45, and providing treatment for 90% of identified precancerous lesions (18). However, current screening coverage remains suboptimal, particularly in low- and middle-income countries – a global study covering 202 countries showed that two-thirds of women aged 30 to 49 have never been screened for cervical cancer, and these regions also bear the highest disease burden (9). In terms of screening strategy, the HPV genotyping-based approach holds significant value. Research indicates that HPV genotyping can enable approximately 13.2% of those screened to qualify for an accelerated treatment pathway (38), and it is comparable in safety to the traditional multiple-visit colposcopy follow-up model while requiring fewer screening visits, making it particularly suitable for resource-limited settings (39). Furthermore, in response to the observed resurgence in infection rates among women aged ≥61 years identified in this study, it is essential to emphasize the importance of continuous screening until age 65, stopping only after meeting specific exit criteria (30). Effective screening and treatment are expected to significantly reduce the HPV-related disease burden in this age group (36).

In summary, to comprehensively address the dual-peak challenge of HPV infection, it is necessary to promote adolescent vaccination to control the first peak while simultaneously strengthening screening coverage and standardization across all age groups – particularly among middle-aged and older women – to establish a dual prevention system of “vaccination and regular screening.” With the widespread adoption of HPV vaccination and the promotion of HPV-based screening strategies, cervical cancer has the potential to become a rare disease in the coming decades (40).

Of the 11,360 HPV-positive cases in this study, single infections were predominant (76.47%), while multiple infections accounted for 23.53%. This distribution pattern is highly consistent with previous studies, which typically report single infection rates ranging from 70 to 80% and multiple infection rates between 20 and 30% (21–26). Further analysis revealed that mixed high-risk and low-risk (HR + LR) infections accounted for 12.29% of all HR infections (including single HR infections and HR mixed infections) and 39.40% of all LR infections (including single LR infections and LR mixed infections). These findings have clear clinical implications: assuming all HR types have carcinogenic potential and all LR types primarily cause genital warts, clinicians managing a cervical cancer patient have a probability of approximately 12.29% of detecting a concurrent low-risk HPV infection. More importantly, conversely, when diagnosing a patient with genital warts, there is a significantly higher probability of approximately 39.40% of concurrently detecting a HR HPV infection. Therefore, it is strongly recommended that clinicians perform HPV genotyping before and after treating patients with genital warts (particularly prior to procedures such as excision) to accurately identify and assess potential mixed infections with HR types, thereby enabling more comprehensive risk evaluation and patient management.

The occurrence of single or multiple HPV infections in women and their associated risks is a complex issue involving multiple factors. On one hand, viral factors are evident: different HPV subtypes can infect the cervical mucosa through shared pathways, and the inherent infectivity of each genotype varies—this study confirms that certain high-risk types (e.g., HPV-52 and HPV-16) dominate in both single and multiple infections. On the other hand, host factors are critical, as an individual’s immune status and genetic susceptibility determine their risk of infection with specific HPV types (41). Furthermore, epidemiological studies indicate that the probability of multiple infections is higher among young, sexually active women with multiple sexual partners, and their subsequent risk of developing related diseases is significantly increased (42). Regarding carcinogenic risk, there is still no consensus in the academic community. Some studies have shown that, compared to single infection, multiple infections can increase the risk of cervical cancer by up to 11.9 times and are considered an important risk factor for high-grade cervical intraepithelial neoplasia (CIN2+) (43, 44). However, other viewpoints suggest that the severity of lesions depends primarily on the inherent carcinogenic potential of the high-risk types involved, rather than on the number of types infected (45). It is worth noting that potential biological interactions among different HPV types in multiple infections, as well as differences in the host immune microenvironment, may contribute to the inconsistency in research conclusions. In summary, whether there is an essential difference in carcinogenic risk between single and multiple HPV infections still requires further investigation and validation through more refined molecular epidemiological studies and long-term follow-up data.

This study has several limitations. First, as a retrospective analysis conducted at a single medical center with participants primarily consisting of women seeking healthcare services, potential selection bias exists, and caution is warranted when generalizing the findings to the general female population in Chengdu. Second, the lack of key behavioral data (e.g., detailed sexual history) and HPV vaccination history limited further in-depth attribution analysis of the observed infection patterns and trends. Furthermore, this study did not correlate HPV infection status with cervical cytological or histopathological outcomes, thus precluding assessment of the association between various infection statuses and the subsequent risk of developing clinical lesions.

## Conclusion

In this study, although the overall HPV prevalence rate (22.03%) showed a significant decrease compared to historical data from 2013 to 2020 (23.28%; *χ*^2^ = 35.065, *p* < 0.001), a continuous rebounding trend has been observed since 2020. HPV-52, HPV-16, and HPV-58 were identified as the three predominant high-risk genotypes, dominating the regional HPV epidemiology. A distinctive bimodal age distribution was characterized by a first peak in adolescents and young women aged ≤20 years (49.35%) and a second peak in older women aged ≥61 years (30.79%). Notably, this study is the first in this region to identify significant seasonal variation, with significantly higher HPV prevalence rates in spring, summer, and autumn than in winter. The study revealed that a substantial proportion (39.40%) of LR-HPV infections were actually HR-LR mixed infections. This finding strongly suggests that comprehensive HPV genotyping for patients with genital warts—particularly prior to procedures such as excision—is crucial for clinicians to identify potential high-risk type co-infections, thereby enabling more accurate risk stratification and follow-up management. To address the first infection peak in young women, priority should be given to promoting the 9-valent vaccine covering HPV-16/52/58, especially before sexual debut. To effectively reduce the second infection peak in older women, it is essential to strengthen systematic cervical cancer screening for middle-aged women and maintain screening until at least 65 years of age in accordance with international guidelines. This study has several limitations, including its single-center retrospective design, lack of vaccination and sexual behavior data, and failure to integrate cytological or histopathological results to assess disease progression risk. To effectively reduce the cervical cancer burden in Chengdu and similar regions, a dual strategy combining precision vaccination with strengthened screening across all age groups must be implemented.

## Data Availability

The raw data supporting the conclusions of this article will be made available by the authors, without undue reservation.

## References

[1] Muñoz N Castellsagué X de Berrington González A Gissmann L. Chapter 1: HPV in the etiology of human cancer. Vaccine. (2006) 24:1–10. doi: 10.1016/j.vaccine.2006.05.115 16949995

[2] Walboomers JMM Jacobs MV Manos MM Bosch FX Kummer JA Shah KV . Human papillomavirus is a necessary cause of invasive cervical cancer worldwide. J Pathol. (1999) 189:12–9. doi: 10.1002/(SICI)1096-9896(199909)189:1<>3.0.CO;2-F 10451482

[3] Stanley M. Immune responses to human papillomavirus. Vaccine. (2006) 24:6–22. doi: 10.1016/j.vaccine.2005.09.002, 16219398

[4] Zhang JY Zha TZ Wang XM He WJ. The carcinogenicity of human papillomavirus types reflects viral evolution. Virology. (2005) 337:76–84. doi: 10.1016/j.virol.2005.04.002 15914222

[5] Muñoz N Bosch FX de Sanjosé S Herrero R Castellsagué X Shah KV . Epidemiologic classification of human papillomavirus types associated with cervical cancer. N Engl J Med. (2003) 348:518–27. doi: 10.1056/NEJMoa021641, 12571259

[6] Crosbie EJ Einstein MH Franceschi S Kitchener HC. Human papillomavirus and cervical cancer. Lancet. (2013) 382:889–99. doi: 10.1016/S0140-6736(13)60022-7, 23618600

[7] Wang XL Huang XM Zhang YZ. Involvement of human papillomaviruses in cervical cancer. Front Microbiol. (2018) 9:2896. doi: 10.3389/fmicb.2018.02896, 30546351 PMC6279876

[8] Smith JS Lindsay L Hoots B Keys J Franceschi S Winer R . Human papillomavirus type distribution in invasive cervical cancer and high-grade cervical lesions: a meta-analysis update. Int J Cancer. (2007) 121:621–32. doi: 10.1002/ijc.22527, 17405118

[9] Bruni L Serrano B Roura E Alemany L Cowan M Herrero R . Cervical cancer screening programmes and age-specific coverage estimates for 202 countries and territories worldwide: a review and synthetic analysis. Lancet Glob Health. (2022) 10:e1115–27. doi: 10.1016/S2214-109X(22)00241-8, 35839811 PMC9296658

[10] Sung H Ferlay J Siegel RL Laversanne M Soerjomataram I Jemal A . Global Cancer statistics 2020: GLOBOCAN estimates of incidence and mortality worldwide for 36 cancers in 185 countries. CA Cancer J Clin. (2021) 71:209–49. doi: 10.3322/caac.21660, 33538338

[11] ICO/IARC Information Centre on HPV and Cancer. China. Human Papillomavirus and Related Cancers, Fact Sheet. (2023), 2. Available online at: https://hpvcentre.net/statistics/reports/CHN_FS.pdf?t=1701582028479 (Accessed September 15, 2025).

[12] Joura EA Giuliano AR Iversen OE Bouchard C Mao C Mehlsen J . A 9-valent HPV vaccine against infection and intraepithelial neoplasia in women. N Engl J Med. (2015) 372:711–23. doi: 10.1056/NEJMoa1405044, 25693011

[13] Li M Zhao C Zhao Y Li J Wei L. Immunogenicity, efficacy, and safety of human papillomavirus vaccine: data from China. Front Immunol. (2023) 14:1112750. doi: 10.3389/fimmu.2023.1112750, 36993948 PMC10040563

[14] Jit M Brisson M Portnoy A Hutubessy R. Cost-effectiveness of female human papillomavirus vaccination in 179 countries: a PRIME modelling study. Lancet Glob Health. (2014) 2:e406–14. doi: 10.1016/S2214-109X(14)70237-2, 25103394

[15] Pimple SA Mishra GA. Global strategies for cervical cancer prevention and screening. Minerva Ginecol. (2019) 71:313–20. doi: 10.23736/S0026-4784.19.04397-1, 30808155

[16] Shangying H Xiaoqian X Yanyang Z Yawen L Chunxia Y Yueyun W . A nationwide postmarketing survey of knowledge, attitude and practice toward human papillomavirus vaccine in general population: implications for vaccine roll-out in mainland China. Vaccine. (2021) 39:35–44. doi: 10.1016/j.vaccine.2020.11.029 33243631

[17] Zhang M Zhong YJ Wang LM Bao HL Huang ZJ Zhao ZP . Cervical cancer screening coverage China, 2018–2019. China CDC Wkly. (2022) 4:1077–82. doi: 10.46234/ccdcw2022.217, 36751373 PMC9889230

[18] World Health Organization (2020). Global strategy to accelerate the elimination of cervical cancer as a public health problem. Available online at: https://www.who.int/publications/i/item/9789240014107 (Accessed September 10, 2025).

[19] Chen WG Yang CM Xu LH Zhang N Liu XY Ma YG . Gene chip technology used in the detection of HPV infection in esophageal cancer of Kazakh Chinese in Xinjiang Province. J Huazhong Univ Sci Technolog Med Sci. (2014) 34:343–7. doi: 10.1007/s11596-014-1280-6, 24939296

[20] Bruni L Diaz M Castellsagué X Ferrer E Bosch FX de Sanjosé SC . Human papillomavirus prevalence in 5 continents: meta-analysis of 1 million women with normal cytological findings. J Infect Dis. (2010) 202:1789–99. doi: 10.1086/657321 21067372

[21] Zhang JY Zha TZ Wang XM He WJ. Prevalence and genotype distribution of HPV infections among women in Chengdu, China. Virol J. (2024) 21:52. doi: 10.1186/s12985-024-02317-x, 38429823 PMC10908056

[22] Yan X Shen L Xiao Y Wang Q Li F Qian Y. Prevalence, characteristis, and distribution of HPV genotypes in women from Zhejiang Province, 2016–2020. Virol J. (2021) 18:208. doi: 10.1186/s12985-021-01676-z, 34670576 PMC8527678

[23] Han S Lin M Liu M Wu S Guo P Guo J . Prevalence, trends, and geographic distribution of human papillomavirus infection in Chinese women: a summative analysis of 2, 728, 321 cases. BMC Med. (2025) 23:158. doi: 10.1186/s12916-025-03975-6, 40082952 PMC11907810

[24] Zhang W Guo N Li B Shang E Wang J Zhang M . Prevalence and genotype distributon of human papillomavirus infections in Beijing, China between 2016 and 2020. Virol J. (2023) 20:11. doi: 10.1186/s12985-023-01959-7, 36653807 PMC9847084

[25] Li X Xiang F Dai J Zhang T Chen Z Zhang M . Prevalence of cervicovaginal human papillomavirus infection and genotype distribution in Shanghai, China. Virol J. (2022) 19:146. doi: 10.1186/s12985-022-01879-y, 36096810 PMC9465878

[26] Li S Zhang K Yang L Wu J Bhargava N Li Y . Distribution patterns of human papillomavirus genotypes among women in Guangzhou, China. Infect Agent Cancer. (2023) 18:67. doi: 10.1186/s13027-023-00541-8, 37907979 PMC10617049

[27] Reinberg A Smolensky MH. Biological rhythms and medicine. Berlin: Springer (1983).

[28] Zeng Z Austin RM Wang L Guo X Zeng Q Zheng B . Nationwide prevalence and genotype distribution of high-risk human papillomavirus infection in China. Am J Clin Pathol. (2022) 157:718–23. doi: 10.1093/ajcp/aqab181, 34724029

[29] Chan PKS Cheung T-H Tam AOY Lo KWK Yim S-F Yu MMY . Biases in human papillomavirus genotype prevalence assessment associated with commonly used consensus primers. Int J Cancer. (2006) 118:243–5. doi: 10.1002/ijc.21299, 16032705

[30] Perkins RB Wentzensen N Guido RS Schiffman M. Cervical Cancer screening: a review. JAMA. (2023) 330:547–58. doi: 10.1001/jama.2023.13174, 37552298

[31] Zhou H-L Zhang W Zhang C-J Wang S-M Duan Y-C Wang J-X . Prevalence and distribution of human papillomavirus genotypes in Chinese women between 1991 and 2016: a systematic review. J Infect. (2018) 76:522–8. doi: 10.1016/j.jinf.2018.02.008, 29477803

[32] Feng XJ Hou HL Yu Q Wang JS. Market analysis and countermeasures of cervical cancer vaccine in China. China Biotechnol. (2020) 40:96–101. doi: 10.13523/j.cb.2006054

[33] Wellings K Collumbien M Slaymaker E Singh S Hodges Z Patel D . Sexual behaviour in context: a global perspective. Lancet. (2006) 368:1706–28. doi: 10.1016/S0140-6736(06)69479-8, 17098090

[34] Rodríguez AC Schiffman M Herrero R Wacholder S Hildesheim A Castle PE. Rapid clearance of human papillomavirus and implications for clinical focus on persistent infections. J Natl Cancer Inst. (2008) 100:513–7. doi: 10.1093/jnci/djn044, 18364507 PMC3705579

[35] Gargano JW Park IU Griffin MR Niccolai LM Powell M Bennett NM . Trends in high-grade cervical lesions and cervical Cancer screening in 5 states, 2008-2015. Clin Infect Dis. (2019) 68:1282–91. doi: 10.1093/cid/ciy707, 30137283 PMC6783904

[36] Castle PE Schiffman M Herrero R Hildesheim A Rodriguez AC Bratti MC . A prospective study of age trends in cervical human papillomavirus acquisition and persistence in Guanacaste, Costa Rica. J Infect Dis. (2005) 191:1808–16. doi: 10.1086/428779, 15871112

[37] Passmore JA Morroni C Shapiro S Williamson AL Hoffman M. Papanicolaou smears and cervical inflammatory cytokine responses. J Inflamm. (2007) 4:8. doi: 10.1186/1476-9255-4-8, 17456234 PMC1868022

[38] Sawaya GF Saraiya M Soman A Gopalani SV Kenney K Miller J. Accelerating cervical Cancer screening with human papillomavirus genotyping. Am J Prev Med. (2023) 64:552–5. doi: 10.1016/j.amepre.2022.10.014, 36935166 PMC12036626

[39] Kuhn L Denny L. The time is now to implement HPV testing for primary screening in low resource settings. Prev Med. (2017) 98:42–4. doi: 10.1016/j.ypmed.2016.12.030, 28279263 PMC5578476

[40] Arbyn M Weiderpass E Bruni L de Sanjosé S Saraiya M Ferlay J. Estimates of incidence and mortality of cervical cancer in 2018: a worldwide analysis. Lancet Glob Health. (2020) 8:e191–203. doi: 10.1016/S2214-109X(19)30482-6, 31812369 PMC7025157

[41] Mendez F Munoz N Posso H Molano M Moreno V van den Brule AJ . Cervical coinfection with human papillomavirus (HPV) types and possible implications for the prevention of cervical cancer by HPV vaccines. J Infect Dis. (2005) 192:1158–65. doi: 10.1086/444391 16136457

[42] Vaccarella S Franceschi S Herrero R Muñoz N Snijders PJF Clifford GM . Sexual behavior, condom use, and human papillomavirus: pooled analysis of the IARC human papillomavirus prevalence surveys. Cancer Epidemiol Biomarkers Prev. (2006) 15:326–33. doi: 10.1158/1055-9965.EPI-05-0577, 16492924

[43] Lee SA Kang D Seo SS Jeong JK Yoo KY Jeon YT . Multiple HPV infection in cervical cancer screened by HPVDNAChip. Cancer Lett. (2003) 198:187–92. doi: 10.1016/s0304-3835(03)00312-4, 12957357

[44] Spinillo A Bello BD Gardella B Roccio M Dacco MD Silini EM . Multiple human papillomavirus infection and high grade cervical intraepithelial neoplasia among women with cytological diagnosis of atypical squamous cells of undetermined significance or low grade squamous intraepithelial lesions. Gynecol Oncol. (2009) 113:115–9. doi: 10.1016/j.ygyno.2008.12.037, 19181368

[45] Sandri MT Riggio D Salvatrici M Passerini R Zorzino L Boveri S . Typing of human papillomavirus in women with cervical lesions: prevalece and distribution of different genotypes. J Med Virol. (2009) 81:271–7. doi: 10.1002/jmv.21382 19107962

